# Editorial: Molecular Design and Morphology Control of Semiconducting Polymers for High-Performance Transistors and Photovoltaics

**DOI:** 10.3389/fchem.2022.940253

**Published:** 2022-05-30

**Authors:** Mengmeng Li, Haijun Bin, Tomasz Marszalek

**Affiliations:** ^1^ Key Laboratory of Microelectronic Devices and Integrated Technology, Institute of Microelectronics, Chinese Academy of Sciences, Beijing, China; ^2^ University of Chinese Academy of Sciences, Beijing, China; ^3^ Laboratory of Advanced Optoelectronic Materials, Suzhou Key Laboratory of Novel Semiconductor-Optoelectronics Materials and Devices, College of Chemistry, Chemical Engineering and Materials Science, Soochow University, Suzhou, China; ^4^ Max Planck Institute for Polymer Research, Mainz, Germany

**Keywords:** semiconducting polymers, molecular structure, morphology control, field-effct transistors, photovoltaics

In the late 1970s, the discovery of conducting polymers opened a new era of organic electronics, because of which the Nobel Prize in Chemistry 2000 was awarded jointly to Alan J. Heeger, Alan G. MacDiarmid and Hideki Shirakawa. Compared to their inorganic counterparts, conjugated semiconducting polymers, as well as conjugated small molecules, exhibit unique advantages such as mechanical flexibility, solution processability, low cost and large-area fabrication, with potential applications in next-generation electronic devices such as organic field-effect transistors and organic photovoltaics. Up until now, the performance of polymer electronic devices has reached a charge carrier mobility of over 10 cm^2^/(Vs) in transistors and a power conversion efficiency of over 19% in photovoltaics. This remarkable progress is largely attributed to synthetic chemistry, processing technology, and device engineering.

We are pleased to introduce this Frontiers in Chemistry Research Topic on the “*Molecular Design and Morphology Control of Semiconducting Polymers for High-Performance Transistors and Photovoltaics*”. This Research Topic includes original research and review articles on molecular design, supramolecular strategy, optoelectronic property and the photophysical behavior of state-of-the-art organic semiconductors, as well as their functionality in electronic devices.

The morphology strategy of binary blending such as bulk heterojunctions is widely utilized to achieve high performance photovoltaics and light-emitting diodes. It is noteworthy that this method can also be used to investigate charge carrier transport in field-effect transistors. Janus et al. deposited n-type naphthalene diimide (NDIC8) films through spray coating, which was further doped with a small amount of p-type polythiophene (P3HT). They found that in the presence of 2.4 wt% P3HT the NDIC8 was recrystallized from the isotropic crystal structure to a new but not-yet-reported polymorph during solvent vapor annealing. Although the addition of P3HT led to the ambipolar transport in transistors, the corresponding electron mobility was dramatically reduced by around 1 order of magnitude due to the change in the crystal structure.

For conjugated small molecules, cocrystal engineering, which was first reported for tetrathiafulvalene/7,7,8,8- tetracyanoquinodimethane (TTF/TCNQ) pair, is another advanced “binary” supramolecular strategy. Jiang et al. summarized recent advances in organic cocrystals and their functional properties. The first challenge was how to grow defect-free organic cocrystals, which could be realized by the liquid-phase, vapor-phase, and solid-phase methods. With the reorganization of crystal structure, new properties and functionalities were often observed for cocrystals. For instance, both experimental and theoretical results demonstrated that the new organic crystals by assembling donor and acceptor molecules exhibited enhanced optoelectronic properties including ambipolar transport, photoelectric conversion and photoresponse, rivaling or even surpassing the best single-component crystals. More importantly, this review article also discussed the magnetic properties of organic cocrystals, with special attention paid to the multiferroic crystals composed of two semiconductor materials, exploring the potential applications in magnetic-field sensors and memories. It has to be emphasized that the internal mechanism of the magnetic property of organic cocrystals is still poorly understood, and more efforts are required.

The excited-state decay of organic semiconductors critically affects the device performance of organic photovoltaics. Menšík et al. systematically investigated the kinetics of the photoexcited states in thin films of metallo-supramolecular polymers made of bis(terpyridine-4′-yl)terthiophenes and Zn^2+^ coupling ions. Upon excitation at 440 nm, the decay of formed singlet excitons was found to be dependent on pump fluence, demonstrating the exciton-exciton annihilation nature. The related theoretical model indicated that such bi-molecular annihilation was mainly dominated by Förster transfer between singlet states. At the same time, triplet excitons were also generated through singlet fission of higher excited singlet states, where the resultant diffusion coefficient showed power-law time dependence with the reduced diffusivity. Yu et al. also explored the photophysical behavior of organic semiconductors. A chiral difluorenol, DOHSBF, was selected as the model compound, and three diastereomers were obtained, determined from NMR measurements. Interestingly, all diastereomers as well as the mixture exhibited excellent spectral stability (blue emission) without affecting their photoluminescence spectra, facilitating high-performance organic photonics.

Pyrrole is a widely used monomer for constructing functional conjugated molecules in organic electronics. Gao et al. rationally designed and synthesized two new pyrrole-based conjugated microporous polymers by one-step oxidative self-polycondensation. Their large specific surface areas (large pore size) and good stabilities resulted in high catalytic activity toward Knoevenagel condensation reaction, and superior recyclability was also observed. This work largely extended the functionality of conjugated systems.

This Research Topic provides a diverse impression of the highly interdisciplinary nature of organic semiconductors, from synthetic chemistry, materials science, processing technology, device physics and theoretical simulation to their emerging applications such as field-effect transistors and photovoltaics. We greatly appreciate the contributions of all the authors included in this Research Topic, and acknowledge Frontiers in Chemistry for providing this excellent platform for the community working with organic electronics, as well as their editorial support. Last but not the least, we are confident that the concepts and knowledge provided by this Research Topic will appeal to a broad readership of Frontiers in Chemistry.

